# Virtual Reality Based Interventions Among People With Dementia: Applications and Health-Related Outcomes

**DOI:** 10.1093/geroni/igaa057.639

**Published:** 2020-12-16

**Authors:** Yu-Ping Chang, Yanjun Zhou, Hanbin Zhang

**Affiliations:** 1 University at Buffalo, Buffalo, New York, United States; 2 University at Buffalo, Buffalo, United States

## Abstract

The use of virtual reality (VR) technology to improve health among older adults has been receiving increased attention. VR technology has many applications and can produce benefits for people with various medical conditions such as symptom reduction or improving the diagnostic process. Despite the increase in the number of research studies of VR technology, little is known about how it has been used to improve health-related outcomes among people with dementia. This systematic review aimed to synthesize research evidence regarding the scope and impact of VR based interventions among people with dementia. Five databases, CINAHL, Embase, PsychINFO, PudMed, and Web of Science were searched to identify studies leveraging VR to facilitate interventions designed for people with dementia. Multiple keywords were used in combination including: dementia, Alzheimer’s, VR, virtual reality, and Virtual Reality Exposure Therapy. Articles were screened if they were published between 2000 through 2020. Fifteen studies met the criteria, with ten utilizing a randomized controlled trial. The VR based interventions in those studies included virtual physical exercises, virtual forests or natural landscapes, and virtual basic living activities. The results of these studies show that VR as a display medium can greatly improve decision-making, hearing, vision, motor ability, and memory in people with dementia residing either in the community or in long term care settings. Our review demonstrated that VR showed positive benefits through various applications for people with dementia. Future research is needed to make VR interventions more customized toward effectively meeting the needs of people with dementia.

